# Neuroendocrine Tumor of the Fallopian Tube and Serous Adenocarcinoma of the Ovary: Multicentric Primary Tumors

**DOI:** 10.5146/tjpath.2022.01589

**Published:** 2023-05-15

**Authors:** Nataliia Hyriavenko, Mykola Lyndin, Vladyslav Sikora, Ruslana Chyzhma, Yulia Lyndina, Kateryna Sikora, Wireko Andrew Awuah, Anatolii Romaniuk

**Affiliations:** Department of Pathology, Sumy State University, Sumy, Ukraine; Department of Morphology, Sumy State University, Sumy, Ukraine; Department of Student, Sumy State University, Sumy, Ukraine

**Keywords:** Neuroendocrine tumor, Fallopian tube, Serous adenocarcinoma, Ovary, Multicentric primary tumors

## Abstract

Neuroendocrine tumors (NETs) comprise a large group of tumors that are most often localized in the gastrointestinal tract and lungs. They are rarely found in the organs of the female reproductive tract; such NETs are primarily localized in the ovaries. We present a case of multicentric primary low-grade NET of the fallopian tube and high-grade ovarian serous adenocarcinoma. In both tumor regions, the histotypes of neoplasms were determined by morphological and immunohistochemical investigations. The NET of the fallopian tube was diffusely positive for chromogranin A and CD56, but wild type for p53 and negative for CK7, CK20, and ER; Ki-67 expression was observed in 3% of the neoplastic cells. The ovarian serous adenocarcinoma was positive for CK7 and ER, mutant for p53, but negative for chromogranin A, CK20, and CD56; Ki-67 expression was observed in 45% of the tumor cells. These results support the possibility that NET can occur in the female reproductive tract and coexist with other malignant tumors.

## INTRODUCTION

Neuroendocrine tumors (NETs) comprise a large group of tumors that are characterized by biodiversity and heterogeneity. They are mostly localized in the gastrointestinal tract and lungs; however, there are a few reports of diffuse endocrine system tumors in the female reproductive tract, which are primarily localized in the ovaries (1–3). Primary NETs of the fallopian tube are very rare. A few reported cases describe primary neuroendocrine cancers (NECs) of the fallopian tube (4–8). There are only three reported cases of carcinoid, one of which arose from a teratoma (9). In one case, the fallopian tube was affected by a heterogeneous tumor (NEC and serous carcinoma) (10). Due to the low incidence of NETs in the female reproductive organs, it may be difficult to make a diagnosis without additional investigations of the tumor tissue. The presence of multiple primary tumors significantly affects the course of the disease, its treatment, and the survival rate.

In this report, we present a case of multicentric primary tumors of the female reproductive system consisting of a low-grade primary NET of the fallopian tube and a high-grade ovarian serous adenocarcinoma.

## CASE REPORT

A 58-year-old postmenopausal (five years) woman visited the gynecologist with complaints of right-sided lower abdominal pain for the past one month. Physical examination identified a mass in the right ovary. Ultrasound examination revealed a 12.0×7.0×6.0 cm tumor in the right ovary. Blood Ca125 level was elevated (64.82 U/ml). There were no pathological changes on computed tomography of the chest and abdominal cavities. Fibrogastroduodenoscopy revealed antral gastritis. General clinical and biochemical analysis of the blood was without abnormalities. Surgery—panhysterectomy with omentectomy— was performed. Macroscopic examination of the postoperative specimens showed a normal-sized uterus, retention cysts in the uterine cervix, thickening of the endometrium (1.1 cm), an endometrial polyp (0.7×0.5×0.5 cm) in the left uterine corner, and no abnormalities in the left adnexa (only age-related changes). There was a yellow-pink colored, node-shaped tumor measuring 3.0×2.0×1.6 cm in the ampulla of the right fallopian tube. Rest segments of fallopian tube were without visible chances. In the right ovary, a multi-chambered cyst measuring 12.0×7.0×6.5 cm with cloudy fluid and light gray papillae on the inner surface was observed. There was no pathology in the pelvic lymph nodes or omentum. The abovementioned changes were considered to be a malignant tumor of the right ovary with metastases into the wall of the right fallopian tube.

**Figure 1:**
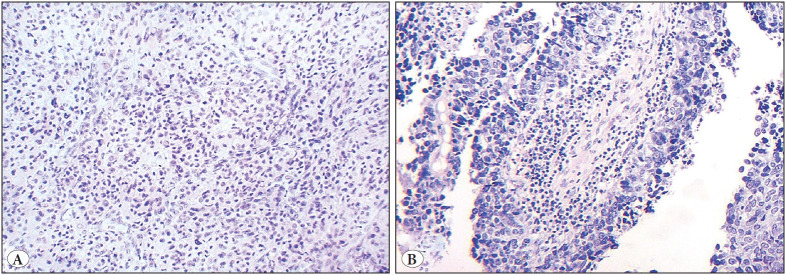
Fallopian tube **(А)** and ovarian tumors **(В).** Hematoxylin and eosin staining (Magnification: ×200).

Morphological analysis revealed retention cysts in the uterine cervix and simple hyperplasia without atypia. An endometrial polyp was diagnosed. The tumor in the right fallopian tube spread into the mucous and submucous membranes without epithelial involvement, as well as metastases in the lymphatic and blood vessels. It had a well-organized histological structure characterized by nests, anastomotic chains, and structures, such as peripheral “palisades” and “rosettes”, of the same type of cells with finely granular (salt and pepper) nuclear chromatin (Figure 1). Mitotic count was 1 mitosis/2 mm2. Histological features of a germ cell tumor (teratoma) and a precancerous lesion in the tubal epithelium in the affected fallopian tube were not found. Concurrently, the right ovarian neoplasm had short (with dense fibrous stroma) and long thin papillae, as well as slit-like glandular structures. They were lined by single- and multilayered columnar epithelium with signs of atypia (large polymorphic and hyperchromic nuclei, prominent large and eosinophilic nucleoli, numerous mitoses, some of which are atypical). In some areas, the tumor formed papillary structures and cell clusters without stroma. Psammoma bodies were observed rarely in the tumor (Figure 1).

**Figure 2:**
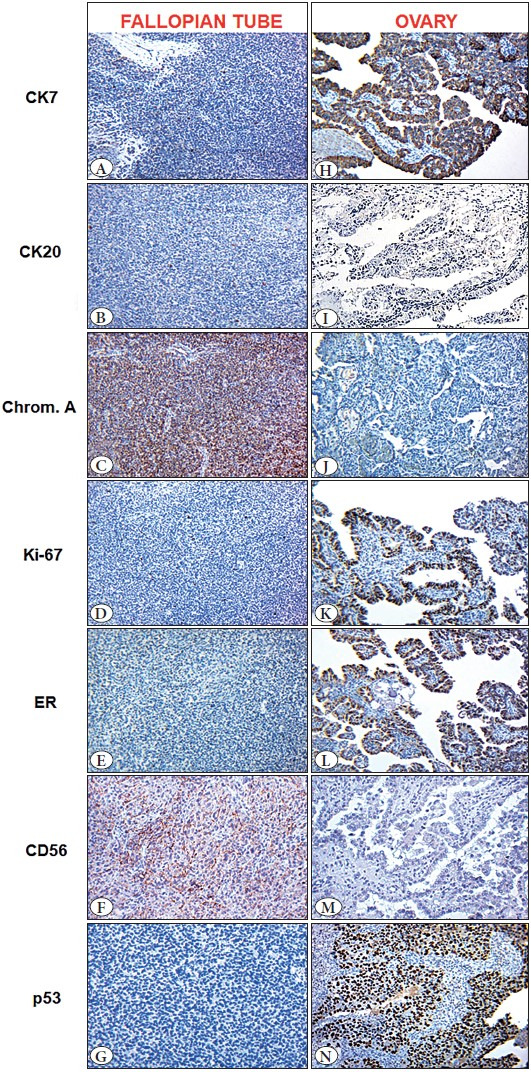
Neuroendocrine tumor of the fallopian tube and ovarian papillary serous adenocarcinoma. Immunohistochemical staining: CK7, CK20, Chromogranin A, Kі-67, ER, CD56, p53. Chromogen – diaminobenzidine; nuclei were counterstained with Mayer’s hematoxylin (Magnification: ×200).

An immunohistochemical study (IHC) was carried out to determine the histogenesis of the ovarian and fallopian tube neoplasms. The following antibodies were used: chromogranin A (clone DAK-A3), CD56 (clone 1B6), СK7 **(**OV-TL12/30), СK20 (Ks20.8), ER (SP1), CDX2 (EPR2764Y), TTF1 (2F4D8), p53 (SP5), and Kі-٦٧ (SP6). The fallopian tube tumor cells were diffusely positive for chromogranin A and CD56, but wild type for p53 and negative for CK7, CK20 and ER. Ki-67 expression was observed in 3% of the neoplastic cells (Figure 2). The ovarian tumor cells were positive for CK7 and ER, mutant for p53, but negative for chromogranin A, CK20, and CD56. Ki-67 expression was observed in 45% of the tumor cells (Figure 2). Both tumors were negative for CDX2 and TTF1 (not shown). The morphology and immunophenotype of the neoplasms corresponded to primary low-grade neuroendocrine tumor of the fallopian tube (Т١а), ICD-O code-8240/3 and high-grade ovarian serous adenocarcinoma (Т١а), ICD-O code-8461/3. Informed consent for publication purposes was obtained from the patient.

## DISCUSSION

We have presented a clinical case of multicentric tumor lesions in the ovary and fallopian tube. The ovarian tumor had short (with dense fibrous stroma) and long thin papillae, as well as slit-like glandular structures. They were lined by single- and multilayered columnar epithelium with cellular atypia and psammoma bodies. IHC revealed positivity of the tumor cells for СK7, p53, and ER and negativity for chromogranin A, СK20, CD56, CDX2, and TTF1. Moreover, the presence of hormonal changes in the endometrium (endometrial hyperplasia and endometrial polyp) and ER expression in ovarian tumor cells can testify to the estrogen effect in carcinogenesis of serous adenocarcinoma (11,12). These morphological features are specific for high-grade serous adenocarcinoma. Concurrently, the fallopian tube tumor was characterized by the formation of nests, anastomotic chains, and structures, such as peripheral “palisades” and “rosettes”, of the same type of cells with finely granular (salt and pepper) nuclear chromatin. It expressed an immunophenotype typical for NETs (cells were diffusely positive for chromogranin A and CD56). Consequently, the final diagnosis was made as follows: low-grade neuroendocrine tumor of the fallopian tube and high-grade ovarian serous adenocarcinoma.

NETs comprise a heterogeneous group of neoplasms that consist of neuroendocrine cells that can be localized in any human organ. They can produce peptide hormones and biologically active amines. The incidence of NETs has been increasing in recent years, which may become the basis for establishing clear criteria to determine tumor differentiation following appropriate treatment and disease prognosis (13). NETs of the female reproductive system account for 2% of all female reproductive system tumors (14,15). Modern morphological diagnostics continue to develop optimal classification and grading systems of NETs in various localizations, taking into account novel molecular, biological, genetic, and IHC studies. There is an active search for diagnostic parameters, markers for the evaluation of individual prognostic factors, and targets for antitumor therapy.

Modern morphological diagnostics of NETs are based on recommendations from the World Health Organization (WHO) (16). In addition, proposals from the European Neuroendocrine Tumor Society (ENETS), which developed the other TNM system for the classification and identification of NET malignancies of the gastrointestinal tract and pancreas (ENETS; 2006, 2007), are being taken into account. Both are focused on NET prognosis. They are based on numerous morphological parameters, including the level of cellular mitotic activity and presence of necrosis.

Studying the peculiarities of terminology, staging, and grading of malignancies—as proposed by WHO, ENETS, the American Joint Committee on Cancer it was determined that none of the classification systems could be considered universal (16–18). Female reproductive system NETs are classified according to the tumor grading system for gastrointestinal tract NETs (19,20).

Malignant tumors of the fallopian tubes are extremely rare (0.14-1.8% of all female reproductive system tumors) and often misdiagnosed as ovarian or uterine pathology (21–26). Despite their rarity, however, fallopian tube cancers can be the primary source of ovarian and peritoneal tumors (23). Another rare pathology is multicentric primary tumors, which can affect one or more topographic regions without carcinogen clonal relationships (24). NETs rarely occur in the female reproductive system. They can occur in the cervix, ovaries, and endometrium, and particularly rarely in the fallopian tubes (4,20,25–31). Fallopian tube NETs were not included to WHO (4th edition) classification of tumors, however in 2020 neuroendocrine neoplasms are discussed as a separate chapter in the current 5th edition of WHO classification of the female genital tract tumors (31,32). The rarity of NETs in gynecological practice makes it difficult to predict and evaluate their clinical behavior and prognosis (2,14). In recent years, the leading experts have developed a comprehensive dataset of universal IHC markers, which makes it possible to carry out the differential diagnosis between NETs and other tumors and thereby determine the neuroendocrine nature of the neoplasm (15). To grade tumor malignancy, which depends on tumor cell proliferation, the Ki-67 proliferation index should be determined (16,18). The main diagnostic IHC markers for NETs that are recommended for practical use are chromogranin A and synaptophysin (17,29,32,33). Other markers, such as CD56, are sensitive but not very specific (34).

The morphological peculiarities and immunophenotypes of NETs are clearly defined and identifiable among other tumors, including those in the fallopian tubes. However, in some cases, the differential diagnosis of a high-grade NET presents some difficulties and depends on the size of the tumor cells (small or large). Since other high-grade epithelial neoplasms may occur in the fallopian tubes (such as serous or endometrioid carcinoma), it is essential to determine the immunophenotype of poorly differentiated tumors using IHC markers. The important task for the morphologist is to determine whether the fallopian tube NET is a primary lesion or a metastatic lesion from another anatomical region. In this regard, IHC studies are limited because NETs from different organs usually have similar immunophenotypes. Two primary IHC markers that are recommended for the diagnosis of metastasis from a well-differentiated NET when no primary lesion is identified are CDX2 and TTF1. In this case, there was no clinical or radiological evidence of any lesions in other locations. Furthermore, the low-grade tumor in the fallopian tube was negative for CDX2 and TTF1, which led us to believe that it was a primary NET. It is plausible that the neoplasm arose from diffusely scattered neuroendocrine cells, as they are present in the normal epithelium of the female reproductive tract (4). Thus, the final diagnosis and stage of the neoplasms changed - the diagnosis “serous adenocarcinoma of the ovary with metastasis to the fallopian tube (stage 2A)” was replaced with “primary low-grade NET of the fallopian tube with serous adenocarcinoma of the ovary (stage 1A)”. In this case, surgery followed by dynamic observation was chosen; no chemotherapy was required, although it had previously been planned.

Accumulated data on the etiology, pathogenesis, biologi-cal characteristics, and peculiarities of the clinical course of NETs will lead to further improvements in the diagnostic and treatment approaches to these rare malignancies, which have been shown to occur in the female reproductive system. Simultaneously, accurate histopathological diagnosis and determination of the tumor differentiation grade are essential in the selection of appropriate conservative treatment. Although carcinoids are considered benign tumors in many anatomical regions, there is no experience with the clinical behavior of these neoplasms in the fallopian tubes.

## CONCLUSION

Our results prove that it is possible for a primary neuroendocrine tumor to occur in the fallopian tubes and coexist with an ovarian serous adenocarcinoma. The neuroendocrine tumor of the fallopian tube was diffusely positive for chromogranin A and CD56, but wild for p53 and negative for CK7, CK20 and ER; Ki-67 expression was observed in 3% of the neoplastic cells. The ovarian serous adenocarcinoma was positive for CK7 and ER, mutant for p53, but negative for chromogranin A, CK20, and CD56; Ki-67 expression was observed in 45% of the tumor cells. Thus, this case confirms the value of morphologic methods of study, which support the differential diagnosis of fallopian tube and ovary tumors that were previously considered poorly and undifferentiated malignancies.

## Conflict of Interest

The authors report no conflict of interest. The authors declare that the research was conducted in the absence of any commercial or financial relationships that could be construed as a potential conflict of interest.

## Ethical Statements

The study involving human participants was conducted according to the guidelines of the Declaration of Helsinki, reviewed and approved by the Bioethics Committee of Sumy State University (protocol №19/06 from 28/06/2021). The patient provided written informed consent to the publication of the case report results. There are no potentially identifiable human images or data is presented.
